# Urinary continence networks in Parkinson's disease: a resting state functional MRI study

**DOI:** 10.1111/bju.16518

**Published:** 2024-08-27

**Authors:** Holly A. Roy, Christopher Roy, Heidi Tempest, Alexander L. Green, Ricarda A.L. Menke

**Affiliations:** ^1^ Peninsula Medical School University of Plymouth Plymouth UK; ^2^ Derriford Hospital Plymouth UK; ^3^ Department of Urology Churchill Hospital Oxford UK; ^4^ Nuffield Department of Surgical Sciences University of Oxford Oxford UK; ^5^ Department of Neurosurgery John Radcliffe Hospital Oxford UK; ^6^ Nuffield Department of Clinical Neurosciences University of Oxford Oxford UK

**Keywords:** bladder, incontinence, MRI, functional MRI, Parkinson's disease

**Abbreviations**
DBS: deep brain stimulation
FC: functional connectivity
LG: lingual gyrus
PD: Parkinson's disease
rsfMRI: resting‐state functional MRI
STN: subthalamic nucleus

Lower urinary tract symptoms (LUTS) are a common feature of Parkinson's disease (PD), affecting up to 70% of patients [1] and significantly impairing quality of life. Ongoing use of continence aids also represents an economic burden. Deep brain stimulation (DBS) of the subthalamic nucleus (STN) has been shown to improve LUTS in PD [2] and to have a significant benefit regarding improvement of LUTS compared with DBS of the globus pallidus interna [3]. Two positron emission tomography studies that compared brain activity in PD patients during bladder filling with STN DBS ‘ON’ and ‘OFF’ showed that STN DBS enhanced responses to bladder filling in the insula and posterior thalamus [4], implying improved sensory processing, and reduced blood flow in areas associated with urinary urgency, such as the anterior cingulate cortex and the left lateral frontal cortex [5]. As catheterisation and concomitant brain imaging are not always possible in frail patients with PD, we wanted to understand whether an alternative approach, using resting‐state functional MRI (rsfMRI) in patients across two different conditions (full and empty bladder) would be an effective tool to investigate the neural mechanisms underlying urinary symptoms in PD. In order to further explore the mechanism by which the STN is involved in sensory aspects of bladder control circuitry, we undertook an rsfMRI study in DBS‐naive PD patients. Our rationale was that, by understanding the wider brain network changes underlying the role of STN DBS on urinary symptoms, we might reveal further avenues for improved treatment of urine storage problems in PD. Our aim was to compare functional connectivity (FC) between the STN and global brain networks in the empty and full bladder state and thus infer the potential role that the STN may play in bladder control.

The study was carried out in accordance with the Declaration of Helsinki, and received approval from the Oxfordshire Research Ethics Committee B (study 09/H0605/62). Eleven DBS‐naive participants with PD (seven male) were recruited to the study from the Oxford Functional Neurosurgery Department (candidates for DBS surgery) and from a network of research‐interested PD patients in the Oxford area (Promise cohort). Results are reported as median (q25–q75). Two participants were excluded from analysis after testing; one due to caffeine consumption and one due to having taken dopaminergic medication on the morning of scanning. The median age of the remaining participants was 60 (57–69) years. The median PD disease duration was 5 (3–6.5) years, and the average levodopa equivalent daily dose was 475 (250–538) mg. The median ICIQ MLUTS/FLUTS (International Consultation on Incontinence Questionnaire Male/Female LUTS) score was 12 (8–15). The study participants attended with a full bladder (this was according to their subjective rating; no threshold bladder volume or fixed amount of liquid consumption was set) and were required to withhold their morning PD medications. Participants underwent rsfMRI scans in two bladder states: (1) full and (2) empty bladder. Bladder ultrasonography was performed to confirm a full bladder, and voided volume was recorded (Table S1).

The MRI was performed first in the full bladder state, during which an rsfMRI sequence was run. Participants were instructed to lie still with their eyes closed, and were asked to rate their sensation of bladder fullness according to the following scale (0; no bladder sensation, 1; first sensation of bladder filling, 2; first desire to void, 3; normal desire to void, 4; strong desire to void, 5; maximal bladder capacity) before each scan started. After this, the participants voided to empty their bladder and returned to the scanner. The rsfMRI sequence was repeated. A high‐resolution T1‐weighted structural scan was also performed.

All participants reported a decrease in sensation of bladder fullness between the full and empty bladder scans. The median bladder sensation rating during the full bladder rsfMRI scan was 3.5 (3.5–4) and the median bladder volume was 175 (137–388) mL. During the empty bladder condition, the median bladder sensation rating was 1 (0, 1) and the median bladder volume was 32 (23–78) mL. There was a significant difference in both bladder rating between conditions (Wilcoxon signed‐rank test, *P* = 0.009) and bladder volume between conditions (Wilcoxon signed‐rank test, *P* = 0.02). FC between the bilateral STN and the lingual gyrus (LG) was significantly higher in the empty bladder compared with the full bladder condition (significance threshold set at *P* = 0.05; Fig. 1a). To further explore the role of the LG, we created a bilateral LG seed mask and explored the FC between the LG and other brain voxels in the two bladder states. We found that there was a significant inverse relationship (significance threshold set at *P* = 0.05) describing the FC between LG and a widespread network of areas in the full bladder state compared with the empty bladder state, including the right inferior frontal gyrus, right frontal pole, right frontal operculum, right insular cortex, left inferior frontal gyrus, left frontal operculum, left frontal orbital cortex and left cerebellum (Fig. 1b).

**Fig. 1:**
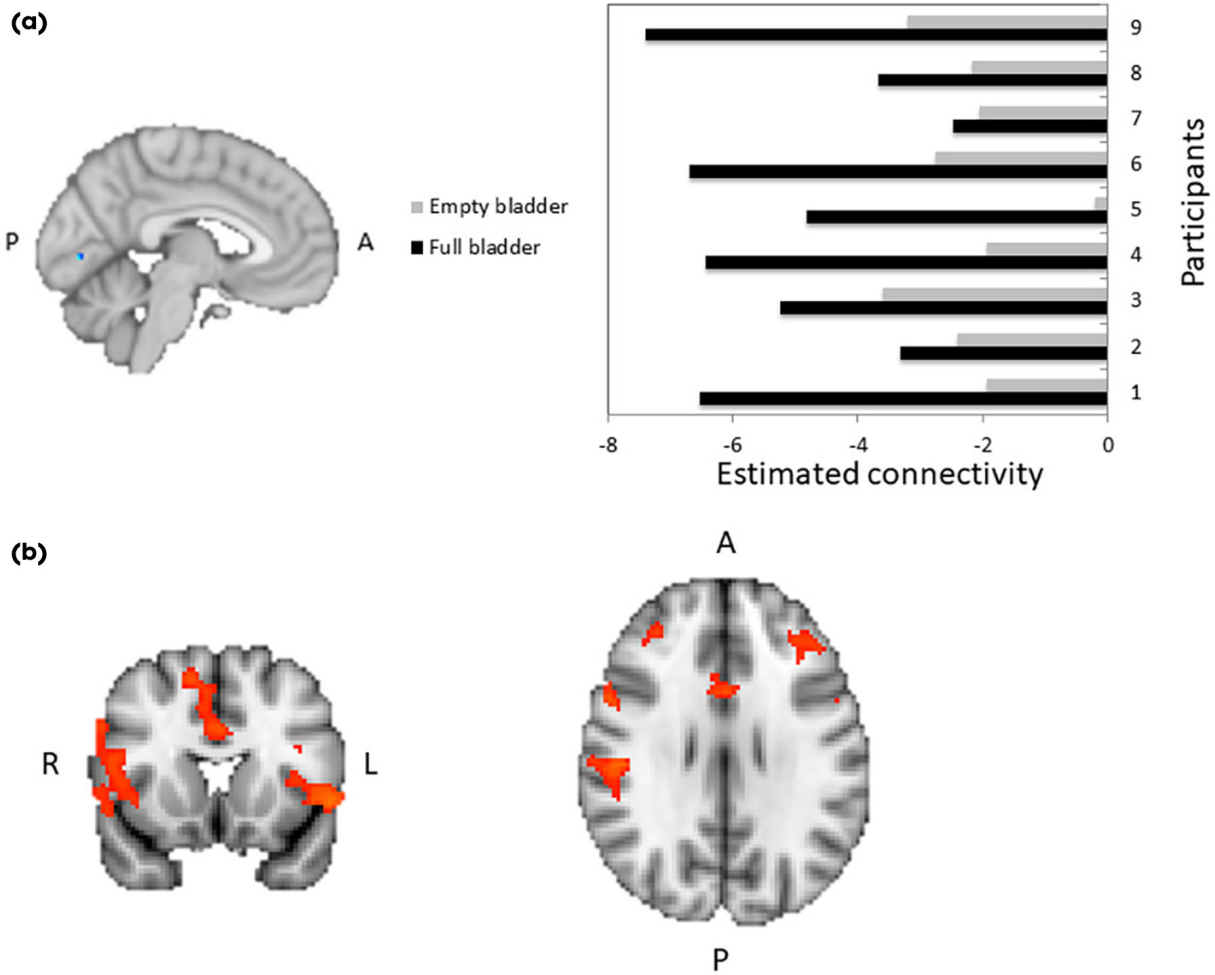
(**a**) Stronger connectivity between lingual gyrus (LG; shown in blue) and subthalamic nucleus in the empty compared with the full bladder condition. (**b**) Significant changes in connectivity between LG mask and widespread brain regions between full and empty bladder states (orange). A, anterior; L, left; P, posterior; R, right.

The STN typically displays pathological hyperactivity in PD due to lack of inhibition from neurons in the substantia nigra. The clinical effect of DBS is thought to result from dampening this activity. Although the primary output of the STN is to the globus pallidus interna and ventrolateral thalamus, rsfMRI studies have identified changes in FC between the STN and widespread brain regions in PD patients compared to controls, including negative coupling between the STN and areas such as the cerebellum and visual cortex [6]. There is also evidence that the STN and LG are connected in an inhibition control‐related neural network that can be impaired by sleep deprivation [7]. Relevant to our findings, the LG and other posterior brain regions have been identified in numerous fMRI studies investigating the brain's response to bladder filling [8], but their role is not yet understood.

In the present study, a significant reduction in correlated activity between the STN and LG occurred in the full bladder state compared with the empty bladder state. Post hoc connectivity analysis using the whole LG as a seed region demonstrated significant increases in FC between LG and multiple areas previously linked with bladder sensation (e.g., insula), urinary urgency (e.g., anterior cingulate cortex) and the response to a full bladder (e.g., frontal regions) in the full compared with the empty bladder state. Overall, these findings may reflect a role of the STN–LG pathway in inhibitory control over the bladder in PD and supports existing evidence that the LG has a functional involvement in the network of brain areas that process information about a full bladder. Further investigations of this effect using a control group of healthy age‐matched participants would help to determine whether the STN–LG connectivity and its role in bladder sensation and continence is specific to PD or is also relevant in healthy subjects. Moreover, targeting the LG using non‐invasive neurostimulation approaches could help to clarify the role of this region in bladder control networks, and may offer potential new avenues for neuromodulation of bladder control networks. A limitation of this study was the absence of urodynamic data on its participants, as these could help differentiate between neurogenic lower urinary tract dysfunction and other causes of LUTS, such as prostate hypertrophy. Future studies should include formal urodynamic assessments in all participants.

In conclusion, this study demonstrated that rsfMRI across bladder states can yield useful information about brain networks involved in continence in PD and highlights the STN–LG connection as an important focus of future investigation.

## Disclosure of Interests

The authors do not have any conflicts of interest to declare.

## Supporting information


**Table S1.** Demographic variables, levodopa dosing, validated questionnaire results and bladder volumes and sensation in the two scan conditions.
