# Supplementary material for: Urinary continence networks in Parkinson's disease: a resting state functional MRI study
Source: BJU Int. 2024 Aug 27;135(1):57–9. doi: 10.1111/bju.16518 (PMC11628875; doi:10.1111/bju.16518)
Supplement: Supplementary file 1 — Table S1. Demographic variables, levodopa dosing, validated questionnaire results and bladder volumes and sensation in the two scan conditions. [file BJU-135-57-s001.docx]

| Patient ID | Sex | Age | Duration of disease | Levodopa equivalent daily dose (mg) | ICIQ total symptom score (FLUTS/MLUTS) | MMSE | Hoehn & Yahr | Full bladder rating | Empty bladder rating | Bladder ultrasound pre-void (ml) | Bladder ultrasound post-void (ml) |
| --- | --- | --- | --- | --- | --- | --- | --- | --- | --- | --- | --- |
| FMRI001 | M | 64 | 8 | 544 | 8 | - | 3 | 3 | 2 | - | - |
| FMRI002 | M | 68 | 12 | 635 | 8 | 29 | - | 3.5 | 1.5 | 384 | - |
| FMRI003 | M | 56 | 2 | 1915 | 15 | 30 | 2 | 4 | 2 | 79 | 6 |
| FMRI004 | M | 71 | 2 | 300 | 14 | 27 | 1 | 3 | 0 | 167 | 32 |
| FMRI005 | F | 57 | 8 | 150 | 12 | 30 | 2 | 4 | 0 | 398 | 69 |
| FMRI006 | M | 59 | 4 | 475 | 6 | 30 | 2 | 3.5 | 1 | 183 | 97 |
| FMRI007 | M | 60 | 3 | 250 | 7 | 30 | 1 | 3.5 | 1 | 735 | 28 |
| FMRI008 | F | 74 | 5 | 505 | 16 | 28 | 2 | 4 | 0 | 148 | 87 |
| FMRI009 | F | 43 | 6 | 100 | 19 | 30 | 1 | 3.5 | 1 | 102 | 18 |

Supplementary table 1.

Demographic variables, levodopa dosing, validated questionnaire results and bladder volumes and sensation in the two scan conditions
